# Cysticercosis in Shandong Province, Eastern China

**DOI:** 10.3201/eid2402.151253

**Published:** 2018-02

**Authors:** Gongzhen Liu, Yanshen Li, Yong Cui, Bingcheng Huang, Hongfa Wang, Yanping Chen, Jingxuan Kou, Fuyong Wang, Chongxing Zhang, Yong Huang, Yin Li, Meng Wang, Qingkuan Wei, Jin Li, Hui Sun, Kun Yin, Guihua Zhao, Yan Jiang, Xin Mao, Zhenhua Yu, Xin Liu

**Affiliations:** Shandong Institute of Parasitic Diseases, Shandong Academy of Medical Sciences, and World Health Organization Collaborating Centre on Vector-Borne Diseases and Food-Borne Parasitic Diseases, Jining, China (G. Liu, Y. Cui, B. Huang, H. Wang, Y. Chen, J. Kou, F. Wang, C. Zhang, Y. Huang, Q. Wei, J. Li, H. Sun, K. Yin, G. Zhao, Z. Yu, X. Liu);; Yantai University, Yantai, China (Yanshen Li, X. Mao);; China Animal Health and Epidemiology Center, Qingdao, China (Yin Li);; Qufu Normal University, Jining, China (M. Wang);; The People’s Liberation Army No. 405 Hospital, Yantai (Y. Jiang)

**Keywords:** epidemiology, cysticercosis, geographical distribution, parasites, epidemiology, Shandong Province, China

## Abstract

We analyzed demographic and clinical data and estimated the incidence of cysticercosis in Shandong Province, China, during 1975–2014. Our analyses showed that a cysticercosis-endemic area is present in Shandong Province, especially in its western regions. Improved surveillance and control are needed to address the elevated risk for cysticercosis in this region.

Cysticercosis is an infection of human tissues caused by a tapeworm parasite, *Taenia solium*, commonly found in pork meat. Patients initially see its symptoms in different areas of the human body as cysts (*1*). Cysticercosis is a major cause of epilepsy in low-income countries and is endemic to countries in Latin America, sub-Saharan Africa, and large regions of Asia, including China and India (*2*). Although *T. solium* tapeworms had virtually disappeared from industrialized countries, increased immigration from cysticercosis-endemic areas has led to a resurgence of cysticercosis in North America, Europe, and Australia (*3**,**4*).

Although cysticercosis is one of the most severe tropical diseases in China, few epidemiologic studies of cysticercosis patients have been performed. We analyzed patients’ demographic and clinical data to estimate the cysticercosis incidence for risk in Shandong Province during 1975–2014.

Shandong Province encompasses 91 counties and 17 major cities. We obtained cysticercosis data from Shandong Institute of Parasitic Diseases, the only professional institution for systematic diagnosis and treatment for cysticercosis in Shandong Province during the study period. Any cysticercosis patient identified in the 17 major cities in Shandong Province was sent to and registered at Shandong Institute of Parasitic Diseases for therapy. A confirmed case was considered on the basis of several criteria that included the following (*5*): 1) surgically removed nodules identified as *Cysticercus cellulosae* by tableting, an incubation test, or histopathologic examination; 2) serum or cerebrospinal fluid positive by immunologic examination; 3) patient history of travel to or residence in a disease-endemic area and a history of tapeworms or contact with tapeworm-infected patients; 4) positive results by computed tomography or magnetic resonance imaging for neurocysticercosis or for B-mode ultrasound for cutaneous muscular or ophthalmic cysticercosis; and 5) diagnosis of cysticercosis supported by clinical symptoms, which could include subcutaneous or muscular nodules, headache, dizziness, epilepsy, or visual disturbance. All cysticercosis cases were recorded in medical records each year. Moreover, the source population for our data represented the total population of Shandong Province.

We calculated the 40-year incidence rate by dividing the number of newly diagnosed cases during the examined time period by the province’s midperiod population (i.e., the 1995 population). In total, 1,952 cysticercosis case-patients were identified. The crude 40-year incidence rate was, therefore, 22.4 (95% CI 21.4–­23.4) cases per 1 million population. 

We further calculated incidence rates by age, sex, and residence. Of the 1,952 case-patients, 1,288 (66%) were male and 664 (34%) were female, and more patients lived in rural areas (69.0%) than in urban areas (31.0%). Study data indicated a higher incidence rate for male (29.1 [95% CI 27.5–30.7] cases/1 million population) than female (15.5 [95% CI 14.4­­­­­–­­­­­­16.7]) patients and for rural residence (27.9 [95% CI 25.7–30.1]) than for urban residence (20.6 [95% CI 19.5–21.7]). For age, we observed the highest incidence rate for the 30–39-year age group (37.2 [95% CI 34.1–40.3] cases/1 million population), followed by the 40–49-year age group (32.7 [95% CI 29.5–35.9]) and the 20–29-year age group (26.6 [95% CI 24.0­–29.1]). The <1–9-year age group had the lowest incidence risk (6.7 [95% CI 5.4–8.1]) (Table).

**Table:** Cysticercosis incidence rates by sex, residence, and age group, Shandong Province, China, 1975–2014

Characteristic	No. (%) patients	Incidence rate, cases/1 million population (95% CI)
Sex		
M	1,288 (65.98)	29.1 (27.5–30.7)
F	664 (34.02)	15.5 (14.4–16.7)
Residence		
Rural	1,346 (68.95)	20.6 (19.5–21.7)
Urban	606 (31.05)	27.9 (25.7–30.1)
Age group, y		
<1–9	94 (4.82)	6.7 (5.4–8.1)
10–19	170 (8.71)	12.5 (10.6–14.3)
20–29	410 (21.00)	26.6 (24.0–29.1)
30–39	546 (27.97)	37.2 (34.1–40.3)
40–49	409 (20.95)	32.7 (29.5–35.9)
50–59	185 (9.48)	26.0 (22.2–29.7)
>60	138 (7.07)	14.3 (11.9–16.7)

We determined dynamic geographic distributions of incidence for 3 ten-year periods (1985–1994, 1995–2004, and 2005–2014) and a combined 30-year period (1985–2014) (Technical Appendix). The period with the highest incidence rates was 1995–2004. An analysis of the geographic distribution of cysticercosis cases revealed the highest incidence risks were in the western areas but not in coastal regions of Shandong Province (*6*).

Other studies have found similarly elevated rates of cysticercosis in Shandong Province (*6**–**8*). Our data highlight several distribution features of cysticercosis in this province, including an increased incidence among men, consistent with findings of a previous report (*8*). However, our data showed elevated incidence in different age groups and regions than the study by Chen et al., in which the 10–29-year age group and middle regions of the province showed the highest incidence rates (*6*).

Our study has a few limitations. First, the long, asymptomatic latent period of cysticercosis affects diagnostic efficiency and age-specific incidence estimates. Second, our data were incomplete because of some missing information for cases we identified. Third, independent confirmation might affect incidence estimates from early in the study period. However, our multidiagnostic approach substantially reduced misdiagnosis rates and increased the efficiency of diagnosing cysticercosis (*9*). 

In summary, our analyses show that Shandong Province has been a cysticercosis-endemic area for many years. Improved surveillance and control are needed to address the elevated risk for cysticercosis in western regions of this province.

Technical Appendix Dynamic geographic distribution of cysticercosis incidence risk in Shandong Province, China, 1985–2014.
