# Supplementary material for: Cysticercosis in Shandong Province, Eastern China
Source: Emerg Infect Dis. 2018 Feb;24(2):384–5. doi: 10.3201/eid2402.151253 (PMC5782879; doi:10.3201/eid2402.151253)
Supplement: Technical Appendix — Dynamic geographic distribution of cysticercosis incidence risk in Shandong Province, China, 1985–2014. [file 15-1253-Techapp-s1.pdf]

Article DOI: https://doi.org/10.3201/eid2402.151253

Cysticercosis in Shandong Province, Eastern China

Technical Appendix

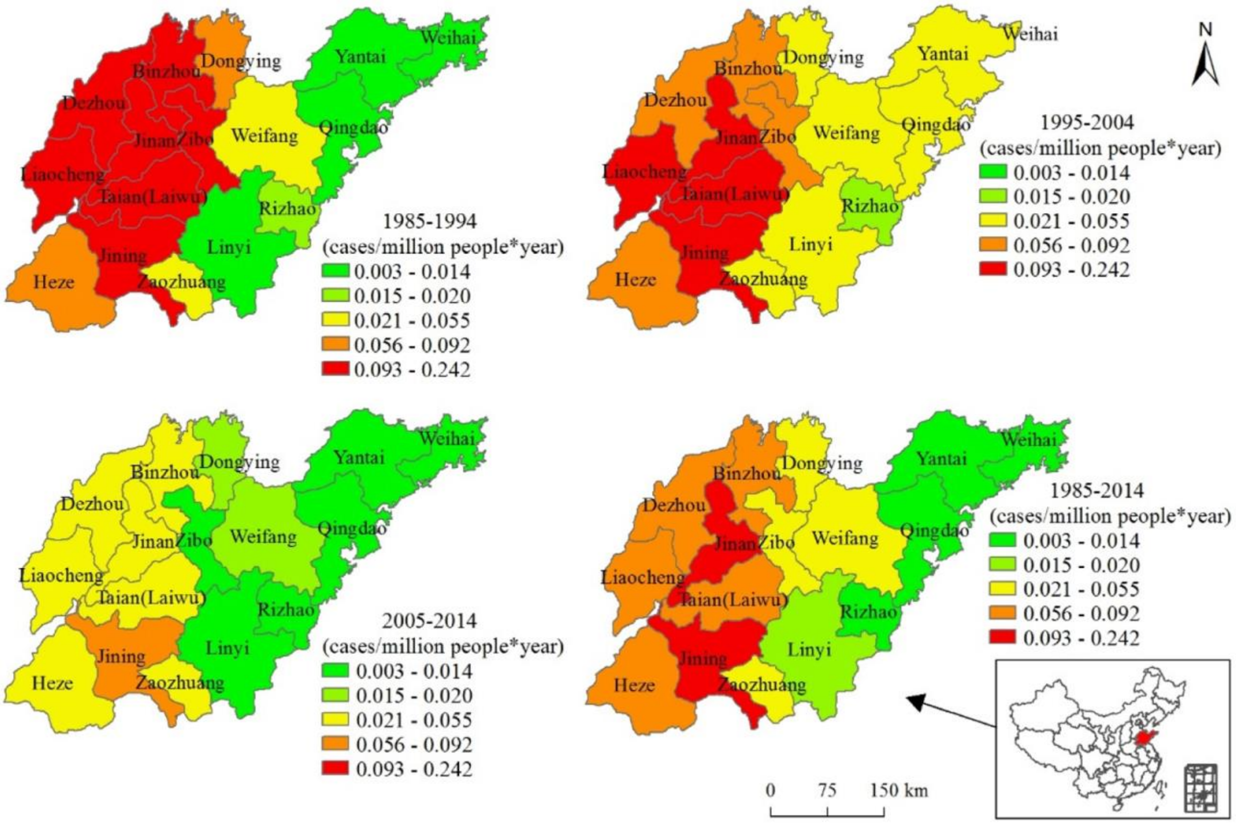

Technical Appendix Figure. Dynamic geographic distribution of cysticercosis incidence risk in Shandong Province, China. A) 1985–1994; B) 1995–2004; C) 2005–2014; D) 1985–2014.
